# Altered Functional Connectivity and Brain Network Property in Pregnant Women With Cleft Fetuses

**DOI:** 10.3389/fpsyg.2019.02235

**Published:** 2019-10-09

**Authors:** Zhen Li, Chunlin Li, Yuting Liang, Keyang Wang, Wenjing Zhang, Renji Chen, Qingqing Wu, Xu Zhang

**Affiliations:** ^1^School of Biomedical Engineering, Capital Medical University, Beijing, China; ^2^Beijing Key Laboratory of Fundamental Research on Biomechanics in Clinical Application, Capital Medical University, Beijing, China; ^3^Department of Radiology, Beijing Obstetrics and Gynecology Hospital, Capital Medical University, Beijing, China; ^4^Department of Oral and Maxillofacial Plastic and Trauma Surgery, Center of Cleft Lip and Palate Treatment, Beijing Stomatological Hospital, Beijing, China; ^5^Department of Ultrasound, Beijing Obstetrics and Gynecology Hospital, Capital Medical University, Beijing, China

**Keywords:** non-syndromic cleft lip and/or palate (NSCLP), pregnant women, resting state functional magnetic resonance (rs-fMRI), functional connectivity (FC), brain network

## Abstract

Non-syndromic clefts of the lip and/or palate (NSCLP) is the most common congenital anomaly in the craniofacial region. NSCLP is a highly gene-associated malformation. We speculate that pregnant women with NSCLP fetuses (pregnancies with NSCLP) may have specific brain changes during pregnancy. To explore characteristic brain function changes of pregnancies with NSCLP, we analyzed resting-state fMRI (rs-fMRI) data of 42 pregnant women (21 pregnancies with NSCLP and 21 pregnancies with normal fetuses) to compare intergroup differences of (fractional) amplitude of low frequency fluctuations (fALFF/ALFF), regional homogeneity (Reho), functional connectivity (FC) and network topological properties. Compared with the control group, increased ALFF in the left hippocampus, the right fusiform and the left anterior cingulate (ACG), increased Reho in left middle occipital gyrus (MOG) and right medial frontal gyrus (MFG) were found for pregnancies with NSCLP. Meanwhile, FC between the left supramarginal gyrus (SMG) and bilateral olfactory cortex (OLF), FC between left precentral gyrus (PreCG) and right MFG, FC between right inferior frontal gyrus (IFG) and left inferior temporal gyrus (ITG) were enhanced in pregnancies with NSCLP. Besides, FC between left PreCG and left amygdala, bilateral para-hippocampal gyrus, FC between left amygdala and left MFG, right IFG were decreased. Graph theory-based analysis explored increased degree centrality (DC), betweenness centrality (BC) and nodal efficiency (Ne) in the left ITG and left SMG for pregnancies with NSCLP. Pregnancies with NSCLP has widespread decreased FC within neural networks of speech and language, which indicated that they were more likely to be associated with defects in speech and language skills. At the same time, increased topological indices showed that speech and language related regions played dominant role in their brain networks. These findings may provide clues for early detection of NSCLP fetuses.

## Introduction

Vast amount of studies on non-human animal studies indicates that during peripartum period, the interplay between pregnancy hormones and peripheral stimulation leads to multiple structural and functional adaptations in the mother’s brain that are necessary for the onset, maintenance, and regulation of maternal behavior (Numan, 2007; Feldman et al., 2019). Researches into peripartum endocrine (Deligiannidis et al., 2013; Hellgren et al., 2013) and immune function (Boufidou et al., 2009; Corwin et al., 2015) in human are increasing, however, studies exploring human brain changes in this period are limited. Structural MRI on healthy primiparous women (first-time mother) explored increase of the pituitary gland (Gonzalez et al., 1988) and widespread reduced gray matter volume (GMV) covering the right temporal lobe, precuneus, prefrontal cortex compared with nulliparous, which was associated with decreased cognitive function (Hoekzema et al., 2017). Recently, Carmona et al. (2019) found a monthly rate of volumetric reductions of 0.09 mm^3^ for both primiparous women and adolescent girls, besides, these reductions were accompanied by decreases in cortical thickness, surface area, local gyrification index, sulcal depth, and sulcal length, as well as increases in sulcal width. There are also studies showing no significant changes in GMV, white matter volume (WMV) or brain volume for primiparous women during this period (Zheng et al., 2018). To our knowledge, there are currently no functional neuroimaging studies during human pregnancy, especially for pregnant women with malformed fetuses. This kind of work might be relevant to understand human brain functional changes and potential neural mechanism during this period and particular situation.

The resting state functional magnetic resonance (rs-fMRI) is an observation of brain spontaneous blood oxygen level-dependent (BOLD) signal changes without stimulation or task status. At present, there are many methods for rs-fMRI analysis, for example, for functional segregation methods, amplitude of low frequency fluctuations (ALFF) analysis is with regards to energy metabolism, which measures the total power of the BOLD signal within the low-frequency range between 0.01 and 0.1 Hz (Zou et al., 2008); Regional homogeneity analysis measures the synchrony of adjacent regions, which calculates the Kendall coefficient of the BOLD time-series of a given voxel and its nearest neighbors (Zang et al., 2004). Previous studies have demonstrated that in most brain areas, there is a significant positive correlation between cerebral blood flow (rCBF) and the regional metabolic rate for glucose (rCMRGlc) and ReHo/ALFF values (Li et al., 2012; Aiello et al., 2015; Nugent et al., 2015). Meanwhile for assessing functional integration features, functional connectivity measures the degree of synchrony of the BOLD time-series between different brain regions, ALFF and ReHo reflect different aspects of regional neural activity (“cities”) but do not provide information on functional connectivity (“highways”) (Lv et al., 2018). Graph theory based-analysis can assess the importance of brain regions at the network level and assess its role in brain information transfer and integration (Rubinov and Sporns, 2010). Thus, these different methods are complementary to each other.

NSCLP is a highly gene-associated malformation. Pregnant women with NSCLP fetuses may have associated risk genes and specific expression, for example, specific brain changes during pregnancy. To explore the characteristic brain function changes of pregnancies with NSCLP, we analyzed their rs-fMRI data and compare their local resting state characteristics, functional connectivity (FC) and brain network topological properties with healthy controls. We speculated that pregnancies with NSCLP would have specific brain function and brain network topological properties, which may provide clues for early detection of NSCLP fetuses.

## Materials and Methods

### Subjects

This study recruited 21 pregnant women with NSCLP fetuses (NSCLP group, 30.76 ± 4.37 years) and 21 pregnant women with normal fetuses (HC group, 31.57 ± 3.01 years) in Beijing Gynecology and Obstetrics Hospital affiliated to Capital Medical University from January to December 2018. Age, educational level and gestation week (GW) of both groups were matched (Table 1). The enrollment criteria mainly include: singleton pregnancy, carrying NSCLP fetus (fetus without other malformations or intrauterine growth restriction, IUGR) or normal fetus. The key exclusion criteria for the two groups are as follows: (1) Brain structural abnormalities including trauma, stroke, tumor or white matter change score ≥ 2 (Wahlund et al., 2001); (2) Neurological or psychiatric disorders, including depression, dementia, and schizophrenia and epilepsy; (3) Physical disability, visual or auditory loss; (4) Complications of pregnancy; (5) MRI contraindications. The research protocol was approved by the Medical Research Ethics Committee of Capital Medical University (Beijing, China). All participants provided written informed consent after being informed of the study details.

**TABLE 1:** Demographic and clinical data of all included subjects.

	**NSCLP (21)**	**HC (21)**	***P***
Age	30.76 ± 4.37	31.57 ± 3.01	0.488^b^
Education	15.0 (11–15.0)	15.0 (11.0–18.0)	0.078^a^
Gestation (week)	25.2 (22.2–31.0)	26.4 (22.5–33.0)	0.191^a^
**POMS**			
Tension	7 (1–23)	3.5 (0–15)	0.600^a^
Anger	4 (0–18)	0.5 (0–13)	0.834^a^
Fatigue	3 (0–16)	2.0 (0–15)	0.972^a^
Depression	3 (0–20)	0.5 (0–15)	0.727^a^
Confusion	6 (1–15)	4.5 (0–19)	0.381^a^
Vigor	10.28 ± 5.85	14.47 ± 6.32	0.059^b^
**IRI**			
PT	13 (8–16)	14 (7–19)	0.465^a^
FS	9.67 ± 2.67	10.11 ± 3.76	0.703^b^
EC	11.89 ± 2.36	12.56 ± 2.45	0.420^b^
PD	11.87 ± 3.04	11.06 ± 3.37	0.477^b^
EPDS	12.07 ± 5.99	9.41 ± 4.53	0.170^b^
AQ	21.57 ± 4.22	23.11 ± 4.63	0.337^b^

*Values distributed normally or non-normally are presented as Mean ± SD or median (minimum, maximum). *P* < 0.05 indicates statistically significant. ^*a*^The Mann–Whitney *U*-test for non-normally distributed data. ^*b*^Two sample *t*-test for normally distributed continuous data (means ± SD). POMS, Profile of Mood States (Shacham, 1983; Chen et al., 2002); IRI, Interpersonal Reactivity Index (Oldfield, 1971); EPDS, Edinburgh postnatal depression scale (Cox et al., 1987); AQ, the adult autism scale (AQ) (Baron-Cohen et al., 2001).*

### Psychological Assessment

Psychological assessment was performed before MRI scanning. Two groups of pregnant women underwent a series of neuropsychological tests, including: the POMS questionnaire (Profile of Mood States) (Shacham, 1983; Chen et al., 2002), the IRI (Interpersonal Reactivity Index), the Edinburgh Handedness Inventory scale (Oldfield, 1971), Edinburgh postnatal depression scale (EPDS) (Cox et al., 1987) and the adult autism scale (AQ) (Baron-Cohen et al., 2001) (Table 1).

### MRI Scan Protocol

MRI scans were performed using a 3.0-T MR scanner (Discovery MR750, GE, Milwaukee, WI, United States). A 32-channel head coil was used to perform the brain scan. Participants were placed on their back in resting state, opened their eyes and remained relaxed during the scan. Earplugs was used to ease the noise and cushion to limit head movement. The parameters of the pregnant woman’s brain scan were as follows: T1-weighted structure image: TR = 8.2 ms, TE = 3.2 ms, flip angle (FA) = 12°, number of excitations (NEX) = 1, field of view (FOV) = 24 cm × 24 cm, imaging matrix = 256 × 256, layer thickness = 1.0 mm, no layer spacing, a total of 164 layers; Resting state function parameters: TR = 2000 ms, TE = 35 ms, FA = 90°, FOV = 24 cm × 24 cm, imaging matrix = 64 × 64, layer thickness = 2.9 mm, no layer spacing, a total of 40 layers.

### fMRI Data Processing

Data pre-processing: Under Matlab2013b, rs-fMRI data was pre-processed using DPARSF version 4 (Chao-Gan and Yu-Feng, 2010). fMRI data were transformed from DICOM into NIfTI format. The first 10 volumes of each participant were removed, slice timing and realignment were performed for the remaining 230 images. Maximum head movement displacement in any direction greater than 2 mm were removed from subsequent analysis and total of 42 participants participated in the following analysis (21 cases in NSCLP group, 21 cases in the HC group); Brain extraction (bet) of the individual T1 structural image was performed before coregister to the functional data. The New Segment module was used to segment the registered images, the DARTEL module was used to create a template which was used to normalize the segmented structural image into the standard Montreal Neurological Institute (MNI) space; the functional images were normalized using the EPI template and resampled into a voxel size of 3 × 3 × 3 mm. Detrend was applied to reduce the systematic drift in the signal, then data was bandpass filtered (0.01–0.10 Hz) to reduce the effects of low frequency drift and physiological noises at high-frequency band (after ALFF and fALFF calculation). Nuisance signal correction was performed with the Friston24 head motion parameters, white matter, and CSF signals and the global signal (Hahamy et al., 2014).

Parameters calculation:

(1) ALFF, fALFF, Reho, seed-based voxel wise connectivity and following Z transformation was calculated with DPARSF version 4.4 to get ALFF map, fALFF map and ReHo map, and formula (each voxel value – whole brain mean)/whole brain standard deviation was used to calculate whole brain Z-transformed ALFF map, fALFF map and ReHo map. Finally, the above brain maps were smoothed by FWHM of 4 mm × 4 mm × 4 mm to obtain images finally used for statistics;
(2) Network topology index and ROI wise FC calculation: Using Gretna2.0.0 (Wang et al., 2015) version, the whole brain was divided into 90 regions of interest (ROI) according to the AAL90 template (Tzourio-Mazoyer et al., 2002), and the time series of BOLD signals in each ROI were extracted. Then, we obtained the Pearson correlation coefficient between the ROIs and calculated the static correlation matrix. The Fisher-Z transformation was performed to obtain the *Z*-value matrix. Two-sample *t*-test was used to calculate the intergroup FC difference; For the *Z*-value matrix of each participant, we did binarization for a given sparsity threshold n%, the strongest connections with the highest n% positive value in the network were set to 1, and the other connections were set to 0. Network sparsity method was used to construct the average brain network of each group, with the sparsity range of 0.05–0.5 and interval of 0.01. The area under the curve (AUC) of each topological value (both global and regional topological metrics) under each sparsity was calculated and the intergroup difference was compared.

For average brain network topological value, in detail, the following parameters were calculated for each participant: The global network metrics included global efficiency (Eglob), local efficiency (Eloc), clustering coefficient (Cp), shortest path length (Lp), and small-world attributes (γ, λ, and σ) (Watts and Strogatz, 1998; Latora and Marchiori, 2001; Onnela et al., 2005; Danon et al., 2006). For the regional network metrics, we evaluated nodal degree centrality (DC), nodal efficiency (Ne), nodal local efficiency (NLe) (Achard and Bullmore, 2007; Buckner et al., 2009; Liang et al., 2013; Liao et al., 2013, 2017). Briefly, Eglob measures the global efficiency of the parallel information transfer in the network and Eloc reveals the network fault tolerance level, which shows the communication efficiency among the first neighbors of a node when it is removed; Cp indicates the extent of local cliquishness in a network and Lp of the network quantifies the mean distance or routing efficiency between any pair of nodes; and small-world attributes indicate the degrees of small-world organization which reflects an optimal balance of integration and segregation for a network. DC reflects the information communication ability; Ne characterizes the efficiency of the parallel information transfer. See Supplementary Material for formulas used to obtain these measurements.

### Statistical Analysis

Demographics, Cognitive Data: Data from two groups were compared using SPSS 22.0 software. Continuous variable normal distribution data were expressed as mean ± standard deviation, analyzed by two-sample *t*-test, non-normal distribution data was expressed as median (range), and Mann–Whitney *U*-test non-parametric test was used. The categorical variables were tested by chi-square test; the above calculations were statistically significant at *p* < 0.05.

Imaging data: Under Matlab 2013b, DPARSF version 4.4 (Chao-Gan and Yu-Feng, 2010) was used to perform two-sample *t*-test on the Z-transformed ALFF map, fALFF map, ReHo map of the two groups. Comparison of ROI wise FC and topological properties were performed with Gretna 2.0.0 (Wang et al., 2015) with two-sample *t*-test. For all between-group analyses, age, gestation week (GW), education level were added as covariates, besides, for local functional parameters (e.g., ALFF), GMV was also added as covariates. Analyses were statistically significant at uncorrected *p* < 0.001 for local functional parameters comparisons, and uncorrected *p* < 0.005 for ROI-wise FC comparisons. To further preserve and explore the differences between the network attributes between pregnancies with NSCLP and normal pregnancies, uncorrected *p* < 0.05 was statistically significant.

## Results

### Intergroup Differences of ALFF, fALFF and ReHo

**FIGURE 1:**
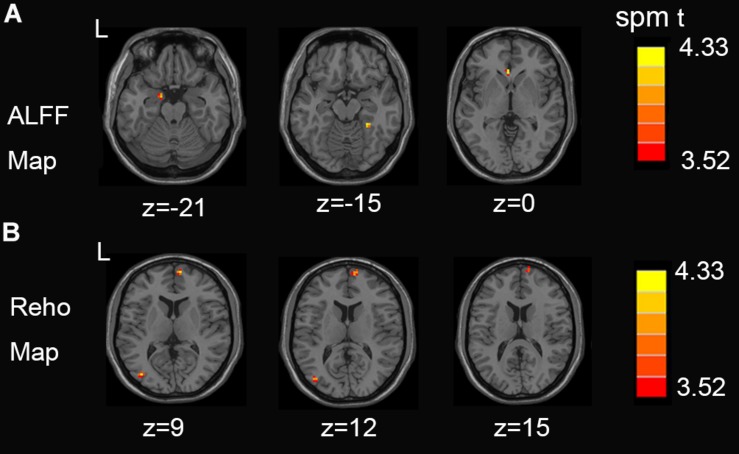
**(A)** Regions exhibiting differences in ALFF between pregnant women with NSCLP and controls (*p* < 0.001, uncorrected). **(B)** Regions exhibiting differences in Reho between pregnant women with NSCLP and controls (*p* < 0.001, uncorrected). No between-group difference of decreased ALFF and Reho was found.

Figure 1 shows between-groups differences in the Z-transformed ALFF and ReHo maps Two-sample *t*-test showed that, compared with the control group, NSCLP group had increased ALFF in the left hippocampus, the right fusiform and the left anterior cingulate (Figure 1A, *p* < 0.001, uncorrected). Increased Reho in the middle occipital gyrus and medial frontal gyrus was found (Figure 1B, *p* < 0.001, uncorrected). The values are shown in Table 2. No between-group difference of decreased ALFF and Reho was found. No statistical between-group difference of fALFF was found either.

**TABLE 2:** Brain regions with significant differences in ALFF, Reho between the NSCLP group and HC group.

	**Brain regions**	**AAL Area**	**Peak MNI coordinates**			***T*-value**	**Cluster size**
			***X***	***Y***	***Z***		
	**NSCLP>HC**						
(A)ALFF	Parahippocampa Gyrus Fusiform Gyrus	Hippocampus_L Fusiform_R	−15 27	−3 −39	−21 −15	4.325 4.521	9 6
	Anterior Cingulate	Cingulum_Ant_L	−3	30	0	4.585	9
	**NSCLP>HC**						
(B)Reho	Middle Temporal Gyrus Medial Frontal Gyrus	Occipital_Mid_L Frontal_Sup_Medial_R	−39 9	−78 63	9 9	4.689 4.557	11 16

*Between-groups differences in the Z-transformed ALFF and ReHo maps (corresponding to Figures 1A,B). MNI, Montreal Neurological Institute; *T*-value of two sample *t*-test (*P* < 0.001 uncorrected). No between-group difference of decreased ALFF and Reho was found.*

### Intergroup Differences of ROI-Wise Functional Connectivity (FC)

**FIGURE 2:**
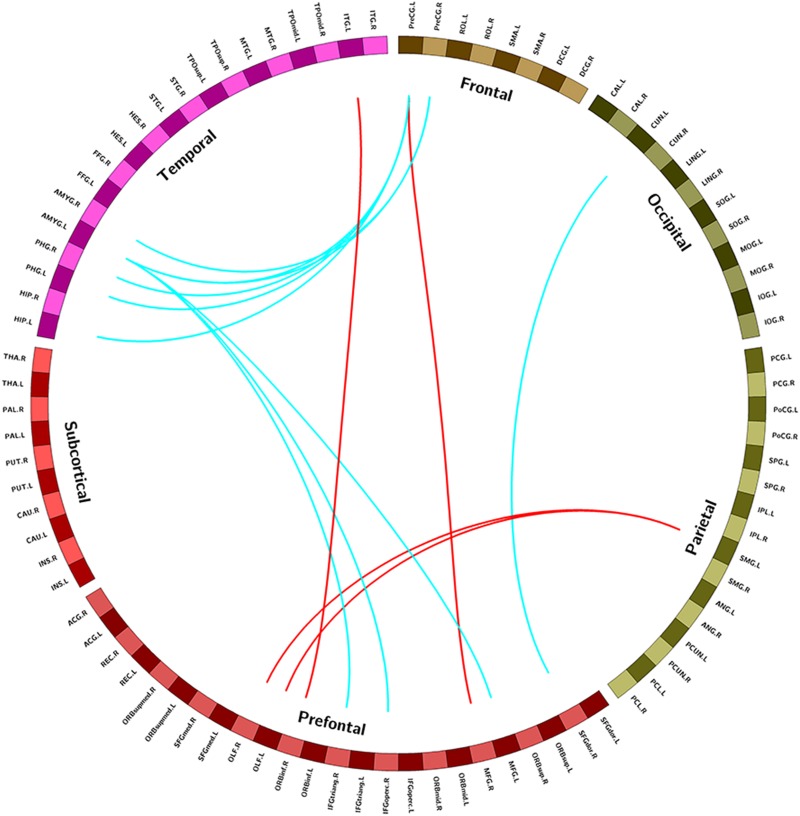
Regions exhibiting intergroup different FC between pregnant women with NSCLP and controls (*p* < 0.005, uncorrected). Brain regions were defined according to the AAL altas (Tzourio-Mazoyer et al., 2002), red edges were increased FC in NSCLP group compared with controls (*p* < 0.005 uncorrected), blue edges were decreased FC in NSCLP group compared with controls (*p* < 0.005 uncorrected).

Compared with the control group, the NSCLP group had widespread altered inter-hemispheric FC mainly in the frontal and temporal regions. In detail, FC between the left supramarginal gyrus and bilateral olfactory cortex, FC between precentral gyrus and middle frontal gyrus, FC between inferior frontal gyrus and inferior temporal gyrus were enhanced in the NSCLP group. Meanwhile, FC between precentral gyrus and amygdala and para-hippocampal gyrus, FC between left amygdala and middle frontal gyrus, inferior frontal gyrus were decreased (*p* < 0.005, uncorrected, Table 3 and Figure 2).

**TABLE 3:** Brain regions with significant differences in functional connectivity between NSCLP group and HC group.

**ROI-based**	**AAL Area**	**AAL Area**	***T*-value**
**connectivity**			
Functional	NSCLP>HC		
connectivity	Precentral_L	Frontal_Mid_R	4.024
	Frontal_Inf_Orb_R	Temporal_Inf_L	3.105
	Olfactory_L	SupraMarginal_L	3.491
	Olfactory_R	SupraMarginal_L	3.776
	NSCLP < HC		
	Precentral_L	Hippocampus_L	−3.466
	Precentral_L	ParaHippocampal_L	−3.303
	Precentral_L	ParaHippocampal_R	−3.343
	Precentral_L	Amygdala_L	−3.456
	Precentral_R	Amygdala_L	−2.986
	Precentral_R	Amygdala_R	−3.296
	Frontal_Sup_R	Cuneus_L	−3.535
	Frontal_Mid_L	Amygdala_L	−3.095
	Frontal_Inf_Oper_R	Amygdala_L	−3.442
	Frontal_Inf_Tri_R	Amygdala_L	−3.998

*A two-sample *t*-test was performed between pregnancies of NSCLP and controls. Age, gestation week (GW), education level served as covariates of no interest. Results are thresholded at *P* < 0.005, uncorrected. Atlas labeling was performed according to the AAL90 atlas (Tzourio-Mazoyer et al., 2002).*

### Intergroup Differences of Topologic Properties

**FIGURE 3:**
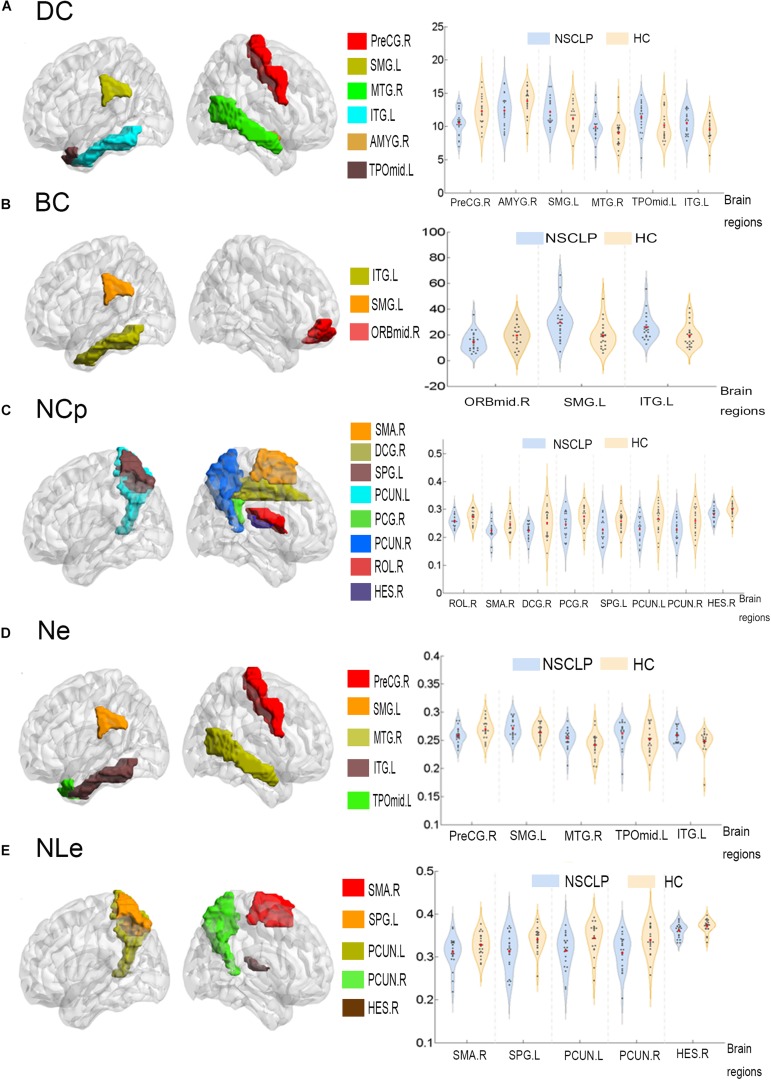
Regions exhibiting intergroup differences of DC **(A)**, BC **(B)**, NCp **(C)**, Ne **(D)**, NLe **(E)** were showed in brain maps on the left, and the intergroup different AUC of DC **(A)**, BC **(B)**, NCp **(C)**, Ne **(D)**, NLe **(E)** were shown in violin pattern for both two groups on the right (*p* < 0.05). BC (betweenness centrality), defined as the fraction of all shortest paths in the network that pass-through a given node. Bridging nodes that connect disparate parts of the network often have a high betweenness centrality. DC (degree centrality) describes the number of connections of a node. It helps identify the highly connected nodes within the network. Ne: The nodal efficiency for a given node characterizes the efficiency of parallel information transfer of that node in the network. NLe: The local efficiency for a given node measures how efficient the communication is among the first neighbors of this node when it is removed. NCp: The local efficiency for a given node was defined as the likelihood that its neighborhoods were connected with each other. PreCG.R, the right precentral gyrus; SMG.L, the left supramarginal gyrus; MTG.R, the right Middle temporal gyrus; ITG.L, the left inferior temporal gyrus; AMYG.R, the right amygdala; TPOmid.L, the left temporal pole: middle temporal gyrus; ORBmid.R, the right middle frontal gyrus, orbital part; SMA.R, the right supplementary motor area; DCG.R, the right median cingulate and paracingulate gyri; SPG.L, the left superior parietal gyrus; PCUN.L, the left precuneus; PCUN.R, the right precuneus; PCG.R, the right posterior cingulate gyrus; ROL.R, the right Rolandic operculum; HES.R, the right heschl gyrus; MTG.R, the right Middle temporal gyrus; SPG.L, the left superior parietal gyrus.

Network topologic property analysis found that, for the global network metrics, there was no significant difference between the two groups in terms of global efficiency (Eglob), local efficiency (Eloc) and small world attribute attributes (γ, λ, σ) (*p* > 0.05, Supplementary Table S1); For the regional network metrics, comparison found that higher degree centrality (DC) (Figure 3A), betweenness centrality (BC) (Figure 3B) and node efficiency (Ne) (Figure 3D) of the left ITG and left SMG in the NSCLP group compared with controls. Besides, DC (Figure 3A) and Ne (Figure 3D) of the left MTG were higher for the NSCLP group (Table 4 and Figure 3). In addition, DC (Figure 3A) and Ne (Figure 3D) of the PreCG in the NSCLP group were reduced (Table 4).

**TABLE 4:** Brain regions with significant differences in topologic index between the NSCLP group and HC group.

**Characteristic**	**AAL Area**	**NSCLP (AUC, mean ± SD)**	**HC (AUC, mean ± SD)**	***P***	***T*-value**
BC	Frontal_Mid_Orb_R SupraMarginal_L Temporal_Inf_L	14.56 ± 7.39 29.61 ± 13.61 25.75 ± 9.77	19.47 ± 8.51 19.45 ± 10.00 19.53 ± 8.80	0.026 0.009 0.043	−2.325 2.774 2.098
DC	Precentral_R Amygdala_R SupraMarginal_L Temporal_Mid_R Temporal_Pole_Mid_L Temporal_Inf_L	10.57 ± 1.88 12.39 ± 2.36 12.17 ± 2.05 9.92 ± 2.07 11.39 ± 2.23 10.57 ± 1.69	12.31 ± 2.18 13.82 ± 1.93 11.26 ± 1.94 8.96 ± 1.98 10.17 ± 2.26 9.58 ± 1.52	0.007 0.039 0.029 0.015 0.018 0.015	−2.836 −2.137 2.273 2.553 2.473 2.560
NCp	Rolandic_Oper_R Supp_Motor_Area_R Cingulum_Mid_R Cingulum_Post_R Parietal_Sup_L	0.26 ± 0.02 0.22 ± 0.04 0.22 ± 0.04 0.25 ± 0.04 0.23 ± 0.04	0.27 ± 0.02 0.25 ± 0.03 0.25 ± 0.05 0.28 ± 0.04 0.26 ± 0.04	0.043 0.017 0.048 0.040 0.018	−2.098 −2.512 −2.045 −2.129 −2.476
	Precuneus_L Precuneus_R Heschl_R	0.23 ± 0.04 0.23 ± 0.04 0.28 ± 0.02	0.27 ± 0.05 0.26 ± 0.05 0.30 ± 0.02	0.004 0.036 0.030	−3.035 −2.182 −2.255
Ne	Precentral_R SupraMarginal_L Temporal_Mid_R Temporal_Pole_Mid_L Temporal_Inf_L	0.26 ± 0.01 0.27 ± 0.02 0.25 ± 0.02 0.26 ± 0.02 0.26 ± 0.01	0.27 ± 0.02 0.26 ± 0.01 0.24 ± 0.02 0.25 ± 0.02 0.25 ± 0.02	0.039 0.010 0.001 0.023 0.003	−2.141 2.709 3.482 2.372 3.170
NLe	Supp_Motor_Area_R Parietal_Sup_L Precuneus_L Precuneus_R Heschl_R	0.31 ± 0.04 0.31 ± 0.04 0.31 ± 0.04 0.31 ± 0.04 0.36 ± 0.02	0.33 ± 0.03 0.34 ± 0.03 0.34 ± 0.04 0.34 ± 0.03 0.37 ± 0.02	0.033 0.017 0.012 0.033 0.035	−2.217 −2.510 −2.639 −2.214 −2.183

*Two-sample *t*-test was performed between pregnancies of NSCLP and controls. Age, gestation week (GW), education level served as covariates of no interest. Results are thresholded at *P* < 0.05, uncorrected. Brain regions with intergroup AUC value of DC, BC, NCp, Ne, and NLe were displayed. Atlas labeling was performed according to the AAL90 atlas (Tzourio-Mazoyer et al., 2002).*

**FIGURE 4:**
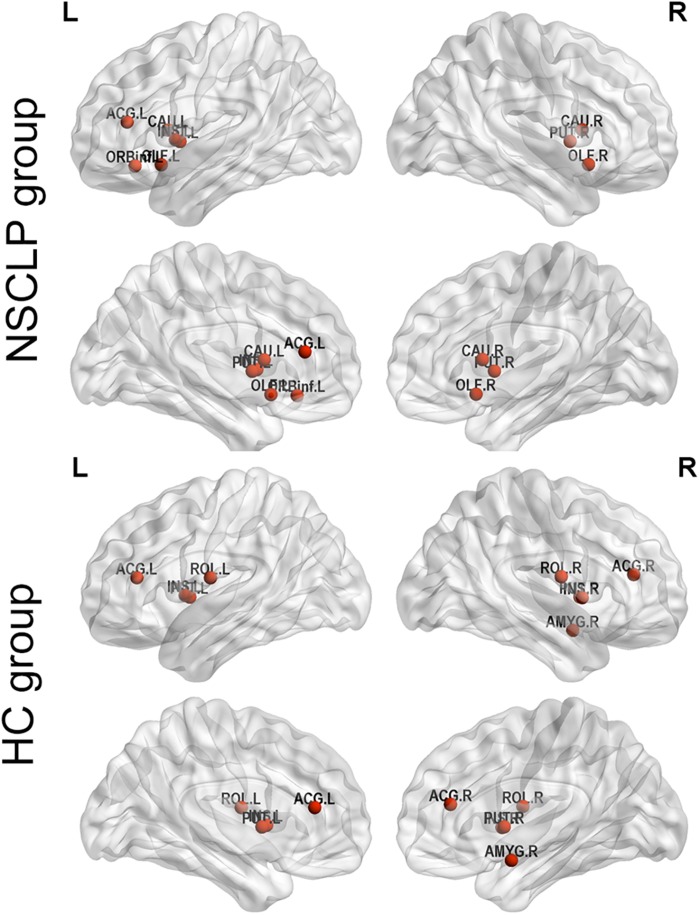
Hub regions of pregnant women with NSCLP fetuses (NSCLP group) and those with healthy fetuses (HC group). Definition of hubs was based on degree centrality (DC) for each node in the previous 10% of the average network for each group. ORBinf.L, the **left** inferior frontal gyrus, orbital part; OLF.L, the **left** olfactory cortex; OLF.R, the **right** olfactory cortex; INS.L, the **left** insula; ACG.L, the **left** anterior cingulate and paracingulate gyri; CAU.L, the **left** caudate nucleus; CAU.R, the **right** caudate nucleus; PUT.L, the **left** lenticular nucleus, putamen; PUT.R, the **right** lenticular nucleus, putamen; ROL.L, the **left** rolandic operculum; ROL.R, the **right** rolandic operculum; ACG.R, the **right** anterior cingulate and paracingulate gyri; AMYG.R, the **right** amygdala.

We define hub nodes based on DC for each node in the previous 10% of the average network for each group (Table 5 and Figure 4). The left and right putamen, the left insula, the right anterior cingulate and paracingulate gyri were their shared hubs. For the NSCLP group, more hubs were in the left hemisphere, the left and right olfactory, the left and right caudate and the left inferior frontal gyrus were their unique hubs, while for the controls, the left and right rolandic operculum, the right amygdala and insula, the right anterior cingulate and paracingulate gyri were their unique hubs (Table 5 and Figure 4).

**TABLE 5:** The AUC value of Hubs’ degree centrality in NSCLP and HC groups.

**Hub regions (AAL Area)**	**Degree centrality (AUC, mean ± SD)**
**NSCLP group**	
Insula_L	13.91 ± 1.81
Caudate_R	13.64 ± 1.75
Putamen_L	13.58 ± 1.62
Olfactory_R	13.56 ± 1.73
Putamen_R	13.46 ± 1.54
Olfactory_L	13.45 ± 1.76
Cingulum_Ant_L	13.45 ± 1.58
Frontal_Inf_Orb_L	13.39 ± 2.48
Caudate_L	13.34 ± 1.90
**HC group**	
Putamen_R	14.31 ± 1.57
Insula_L	14.27 ± 1.79
Putamen_L	14.19 ± 1.66
Amygdala_R	13.82 ± 1.93
Insula_R	13.77 ± 1.97
Cingulum_Ant_L	13.47 ± 1.91
Rolandic_Oper_L	13.26 ± 1.61
Rolandic_Oper_R	13.19 ± 1.52
Cingulum_Ant_R	13.12 ± 1.87

*Definition of hubs was based on degree centrality for each node in the previous 10% of the average network for each group.*

## Discussion

In this study, we analyzed rs-fMRI data and found that, compared to controls, pregnancies with NSCLP had increased ALFF in the left hippocampus, the right fusiform and the left ACG, increased Reho in the left MOG and right MFG. Meanwhile, FC between left PreCG and left amygdala, bilateral para-hippocampal gyrus, FC between left amygdala and left MFG, right IFG were decreased. Graph-based analysis explored increased DC, BC and Ne in the left ITG and left SMG for pregnancies with NSCLP. Pregnancies with NSCLP had widespread decreased FC within neural networks of speech and language, which indicated that they were more likely to be associated with defects in speech and language skills. At the same time, increased topological indices showed that speech and language related regions played dominant role in their brain networks.

### Intergroup Differences of ALFF and Reho

We found increased ALFF in the left hippocampus, the right fusiform and the left ACG, increased Reho in the left MOG and right MFG in pregnant women with NSCLP compared with controls. Hippocampal region and para-hippocampal gyrus are both deep brain areas important to memory (Voss et al., 2017). Portions of the fusiform gyrus are critical for face and word recognition. Recognizing words and faces engages highly specialized sites within the middle fusiform gyrus (Gerrits et al., 2019). These two regions represented distinct but overlapping neural representations for word’s and face’s processing within the fusiform gyrus (Harris et al., 2016). Meanwhile the MOG is a functional visual area. It contains a full map of the visual world (Millington et al., 2017; Raty et al., 2018; Zhang et al., 2018).

Previous studies have demonstrated that in most brain areas, there is a significant positive correlation between rCBF/rCMRGlc and ReHo/ALFF values. Thus, the high regional ReHo/ALFF values indicate high rCBF/rCMRGlc, and low regional ReHo/ALFF values are associated with low rCBF/rCMRGlc (Li et al., 2012; Aiello et al., 2015; Nugent et al., 2015). As mentioned above, fusiform gyrus and occipital gyrus are both related to visual function. The enhancement of regional brain function in the visually relevant brain regions of pregnant women with NSCLP suggests increase in cerebral blood supply and neuronal activity in these related brain regions, which is essential to recognition and understanding of writing language. Although the areas with altered parameters were localized, it can still be used as a specific brain function indicator for pregnant women with NSCLP, providing some clues for future large sample studies.

### Altered FC Within Speech and Language Neural Network for Pregnancies With NSCLP

#### Decreased FC Between the Left Amygdala and the Prefrontal Language System

Our results showed that NSCLP group had decreased FC between left amygdala and the right IFG (the opercular part and triangular part). In the dual neural network model of speech and language, amygdala belongs to the limbic vocal-initiating network of primary vocal motor network (PVMN), which comprises a vocalization region in the anterior cingulate cortex (ACC) [including parts of BA (Brodmann areas) 24, 25, and 32], the hypothalamus, other limbic diencephalic structures (such as the septum and the subcallosal gyrus) (Jurgens and Pratt, 1979; Dujardin and Jurgens, 2005). PVMN controls non-verbal vocalizations in humans (Hage and Nieder, 2016).

The IFG is a region that belongs to the volitional articulatory motor network (VAMN), which also comprises the caudally bordering ventral premotor cortex (area 6, PMv) and ventrolateral primary motor cortex (BA area 4, M1). Its central executive is Broca’s area. In humans, VAMN cognitively controls vocal output. It gains control over articulation by modulating the output of the PVMN. The new VAMN also plays a vital role in establishing semantics and syntax (Hage and Nieder, 2016).

From the above, as our results showed, FC between the dual neural network model of speech and language (VAMN and PVMN) decreased in pregnancies with NSCLP, which indicates that they might have worse coordination between the two networks, and they were more likely to be combined with defects in speech and language system.

#### Increased FC Between the Left ITG and the Orbital Part of Right IFG

In our study, FC between the left ITG and the orbital part of right IFG was increased. ITG and IFG are both involved in semantic processing. BA47 (pars orbitalis) was complemented in Broca’s area, classically comprises BA44 (pars opercularis) and 45 (pars triangularis) in the left hemispheres, which was instrumental for the production, or articulation, of speech and language (Ardila et al., 2017). Activation of the left inferior frontal cortex (lIFC) is the most consistent finding across several fMRI studies aimed at identifying the semantic processing network, more in area BA 47 and BA 45. Higher semantic demands most reliably activate all parts of posterior LIFG, the left middle and posterior MTG, as well as the right posterior IFG. The semantic processing network seems to include at least LIFC, left superior/middle temporal cortex, and the (left) inferior parietal cortex. To some degree, the right hemisphere homologues of these areas are also found to be activated (Hagoort, 2017).

ITG belongs to semantic areas (such as pSTG/MTG/pITG, SMG, AG, and insula). Semantic sentence ambiguity was found to activate the left posterior temporal cortex including the superior temporal sulcus (STS), MTG, and ITG (Rodd et al., 2005; Friederici, 2011). Rs-MRI study showed that ITG in both hemispheres were found to have overlapping connectivity with several subregions of Broca’s complex (including bilateral pars triangularis and left pars orbitalis) (Papathanassiou et al., 2000; Wise et al., 2001; Xiang et al., 2010). As our results showed, FC between the left ITG and the orbital part of IFG, that is, FC within the semantic processing network was increased in pregnancies with NSCLP. This maybe the characteristic brain function connectivity change in pregnancies with NSCLP, too.

#### Decreased FC Between Left PreCG and Bilateral Para-Hippocampal Gyrus and Left Hippocampal

PreCG is a region that belongs to the VAMN, including the facial and laryngeal motor cortex. Recent experiments in monkeys showed that the homolog of the Broca’s area, as well as the premotor and/or primary motor cortices, were all involved in the initiation of volitional calls that have been uttered in response to visual or auditory stimuli (Gemba et al., 1999; Coude et al., 2011; Hage and Nieder, 2013; Fukushima et al., 2014). When syllables were articulated without using the larynx, activation increased in the left primary motor cortex that controls the face, the upper pons, the left planum temporale and the left posterior perisylvian cortex (Paus et al., 1996). Besides, studies on adults with stutter explored increased regional WMV and more GMV in PreCG (Jancke et al., 2004; Beal et al., 2013) and motor areas were over-activated (Brown et al., 2005). For the production of language, motor and premotor regions are relevant, and for language perception, sensory input systems such as the auditory systems for hearing and the visual system for reading must be recruited (Friederici and Gierhan, 2013).

Meanwhile, hippocampal activation has been found to be modulated by familiarity and success of recall for novel lexical items (Davis et al., 2009), previous fMRI studies have shown that the online maintenance of sensory information during a working memory task activates anterior hippocampus (Ranganath and D’Esposito, 2001). Moreover, multivariate fMRI found that para-hippocampal gyrus responded selectively to visual category identity (Diana et al., 2008). Another fMRI study showed that when adults imitated trained and untrained vowel pair vowel, either type of model could be represented in somatomotor, temporal, cerebellar, and hippocampal neural activation patterns during sensorimotor transformation (ST) (Carey et al., 2017). Therefore, hippocampal and para-hippocampal gyrus were both involved in sensorimotor transformation and recalling in pronunciation and speech.

On the other hand, pronunciation is a process of continuous learning and imitation, while speech imitation is a complex process that requires the interaction of both sensory and motor systems, such that acoustic inputs can be processed, transformed to target motor outputs, and articulated as speech (Guenther, 2006; Bohland et al., 2010; Guenther and Vladusich, 2012). Decreased FC between left PreCG and left hippocampal in pregnancies with NSCLP, which indicates that pregnant women with NSCLP might have defects in the neural network of pronunciation imitation.

#### Decreased FC Between the Left Cuneus and the Right Superior Frontal Gyrus

The cuneus (BA 17) receives visual information and it is most known for its involvement in basic visual processing. As the core of the VAMN, vlPFC receives highly processed information from higher-order sensory areas of all modalities (Hage and Nieder, 2016). Neurons in the vlPFC (areas 12/47, 45, 44, and 12 orbital) categorize and maintain communicative signals in working memory to guide goal-directed output. This pathway conveys information from the visual system to the vlPFC passing through the dorsolateral PFC (dlPFC) (Hickok and Poeppel, 2007; Rauschecker and Scott, 2009; Ackermann et al., 2014; Fuertinger et al., 2015). Decreased FC between the left cuneus and the superior frontal gyrus in our results may indicate weakening of information transmission on the visual dorsal stream in pregnancies with NSCLP.

Previous behavior and cognition studies on NSCLP population showed that they were combined with speech and language related deficiencies (e.g., reading, pronunciation), and these defects were associated with brain structural and functional abnormalities. In this study, pregnancies with NSCLP has widespread decreased FC within neural networks of speech and language, which indicated that they were more likely to be associated with defects in speech and language skills. Besides, they had cleft fetuses and as a result they were more likely to carry CLP-associated genes and have abnormal brain findings similar to NSCLP population. Their characteristic FC changes might be due to carrying CLP-associated genes or experiencing genetic mutation themselves. However, gene detection was not involved in this study and this needs to be further explored. Meanwhile, language ability of enrolled pregnant women was not evaluated, which makes impossible to evaluate whether there is a difference in language-related ability between the two groups of pregnant women. This is a limitation of this study and a problem that needs to be further explored in the future.

### Intergroup Differences of Topological Property

#### Dominance of Left ITG and Left SMG in Brain Network of the NSCLP Group

Our results showed increased DC, BC, and Ne of the left ITG in pregnancies with NSCLP. As previously mentioned, the left ITG belongs to semantic areas (such as pSTG/MTG/pITG, SMG, AG, and insula). Structural imaging showed that the left ITG had marked atrophy in semantic dementia (Chan et al., 2001). With fMRI, increased activations in the left posterior ITG were found during handling sentences with lexical ambiguities. Semantic sentence ambiguity was found to activate the left posterior temporal cortex including the STS, MTG, and inferior temporal gyrus (Rodd et al., 2005).

Increased DC and BC of the left ITG and left SMG indicates higher numbers of connections of the nodes in brain networks of pregnancies with NSCLP (including connections connecting different parts of the network), which described higher position of the nodes within their brain network, reflecting higher capability on parallel information processes in brain connectomes of the nodes (Hagmann et al., 2008; Rubinov and Sporns, 2010). Besides, higher Ne prompted higher efficiency of parallel information transfer of these two nodes. This may be characteristic changes of brain networks of pregnancies with NSCLP.

#### Hub Distribution

As for hub distribution, pregnancies with NSCLP had more hubs in the frontal and temporal lobe in the left hemisphere, while for normal pregnancies, hubs were symmetrically distributed. The orbital part of IFG was their unique hub regions, which is near BA 47, complemented in Broca’s area and instrumental for the production, or articulation, of speech and language (Ardila et al., 2017).

From the above, brain regions with more importance in brain functional network of pregnancies with NSCLP belong to speech and language neural network. Combined with the altered FC changes, we speculated that increased importance of left ITG and SMG might be a compensatory change of the brain to ensure the normal function.

## Conclusion

In this study, we analyzed rs-fMRI data and found that, compared with the control group, pregnancies with NSCLP had increased ALFF in the left hippocampus, right fusiform and left ACG, increased Reho in the left MOG and right MFG. Meanwhile, FC between left PreCG and left amygdala, bilateral para-hippocampal gyrus, FC between left amygdala and left MFG, right IFG were decreased. Graph-based analysis explored increased DC, BC, and Ne in the left ITG and left SMG for pregnancies with NSCLP. Pregnancies with NSCLP has widespread decreased FC within neural networks of speech and language, which indicated that they were more likely to be associated with defects in speech and language skills. Meanwhile, increased topological indices showed that speech and language related regions played dominant role in their brain networks. However, this study did not evaluate the language ability of enrolled pregnant women, which make it impossible to evaluate whether there is a difference in language-related ability between the two groups of pregnant women. Besides, no risk gene detection was included in this study. These are limitations of this study, which need to be further explored in the future.

## Data Availability Statement

The datasets generated/analyzed during the current study are not publicly available due to patient privacy and ownership issues. Requests to access the datasets should be directed to the corresponding author.

## Ethics Statement

The studies involving human participants were reviewed and approved by the Medical Research Ethics Committee of Capital Medical University (Beijing, China). The patients/participants provided their written informed consent to participate in this study. Written informed consent was obtained from the individual(s) for the publication of any potentially identifiable images or data included in this article.

## Author Contributions

XZ, QW, and CL designed the research. ZL, CL, QW, XZ, and YL performed the research. QW, YL, KW, RC, and WZ were involved in the clinical assessment. ZL and CL analyzed the data and wrote the manuscript.

## Conflict of Interest

The authors declare that the research was conducted in the absence of any commercial or financial relationships that could be construed as a potential conflict of interest.
