# Supplementary material for: Altered Functional Connectivity and Brain Network Property in Pregnant Women With Cleft Fetuses
Source: Front Psychol. 2019 Oct 9;10:2235. doi: 10.3389/fpsyg.2019.02235 (PMC6795235; doi:10.3389/fpsyg.2019.02235)
Supplement: Supplementary file 1 [file Table_1.doc]

**Supplementary** **Material**

**Methods and Materials**

**Graph-Theory Network Analysis**

We calculated both global and regional network metrics to characterize the overall architecture and regional nodal centrality of the network of both groups as follows.

**i) Global topological metrics**

**Clustering coefficient (Cp):**

The clustering coefficient of a given node *C_i_*, was defined as the likelihood that neighborhoods were connected with each other . We calculated the clustering coefficient as follows:

$$C_{i}=\frac{\sum_{j\text{,}p} a_{ij}a_{jp}a_{pi}}{k{}_{i}(k_{i}-1)}$$

where *k_i_* is the degree of node *i*, *α_ij_*, *α_jp_*, *α_pi_* are 1 or 0 representing whether there is an edge between node *i* and *j* (or node *j* and *p* or node *p* and *i*) or not. The clustering coefficient *C_i_* is zero if this node is isolated or has just one connection. The clustering coefficient *C_p_* of the network *G*, which indicates the extent of local interconnectivity or cliquishness in a network, is the average of the clustering coefficient over all nodes within this network .

**Shortest path length (***L_p_***):**

The path length between node *i* and node *j* is defined as the sum of the edge lengths along this path. The shortest path length *L*_ij_ is the length of the path for node *i* and node *j* with the shortest length. The shortest path length *L_p_* of the network *G*, which quantifies the mean distance or routing efficiency between any given pair of nodes in the network *G*, is computed as follows:

$$L{}_{p}(G)=\frac{1}{N(N-1)}\sum_{i\neq j\in G} L_{ij}$$

where N is the number of nodes in the network G.

**Network global efficiency (Eglob)**

The global efficiency measures the global efficiency of the parallel information transfer in the network *G* , we computed the global efficiency *E_glob_*:


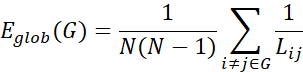


where *L_ij_* is the shortest path length between node *i* and node *j* in the network. N is the number of nodes in the network G.

**Network local efficiency (Eloc):**

The local efficiency of the network *G* measures how efficient the communication is among the first neighbors of nodes *i* when it is removed, we computed the local efficiency *E_loc_*:


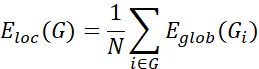


where *G_i_* denotes the subgraph composed of the nearest neighbors of node *i*. N is the number of nodes in the network G.

**Small-world parameters:**

We compared the *C_p_* and *L_p_* of the brain networks with those of random networks to examine the small-world properties in this study. Specifically, we generated 100 random networks with the same number of nodes, edges, weight distribution, and degree distribution as real networks . Then we calculated the *γ* (normalized $C_{p}=C_{p}^{real}/C_{p}^{rand}$) and *λ* (normalized $L_{p}=L_{p}^{real}/L_{p}^{rand}$), where $C_{p}^{real}$ and $L_{p}^{rand}$ are the mean *C_p_* and mean *L_p_* of 100 random networks. The network would be regarded as a small-world network if *σ* > 1 (*σ* = *γ* / *λ*) . Higher σ value indicates a stronger small-world property.

**ii) Regional topological metrics**

**Degree centrality (DC):**

The DC for a given node reflects its information communication ability in the functional network . DC is defined as the sum of all the connections between this node and all of the other nodes in the network.


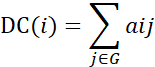


Degree of a node i. *α_ij_* is 1 or 0 representing whether there is an edge between node *i* and *j* or not.

Betweenness centrality (BC):

The nodal betweenness for a given node characterizes its effect on information flow between other nodes. It is defined as the fraction of all shortest paths in the network that pass-through a given node. Bridging nodes that connect disparate parts of the network often have a high betweenness centrality


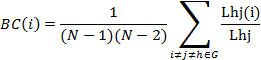


where *L_hj_* is the shortest path length between node *h* and node *j* in the network. *L_hj(i)_* is the number of shortest paths between h and j that pass-through i. N is the number of nodes in the network G.

**Nodal efficiency (Ne):**

The nodal efficiency characterizes the efficiency of parallel information transfer for a given node in the network. *Ne* is measured as the average of the reciprocals of the shortest path length between this node with all other nodes in the network .

**Nodal local efficiency (NLe):**

The local efficiency for a given node measures how efficient the communication is among the first
neighbors of this node when it is removed.

Table S1 Group comparisons of AUC values of global network properties without significant intergroup difference

|  | NSCLP  (AUC, mean±SD) | HC  (AUC, mean±SD) | *P* |
| --- | --- | --- | --- |
| Eglob | 0.26±0.005 | 0.26±0.004 | 0.466 |
| Eloc | 0.34±0.006 | 0.35±0.006 | 0.178 |
| Cp | 0.26±0.013 | 0.26±0.012 | 0.274 |
| Lp | 0.85±0.036 | 0.86±0.035 | 0.541 |
| γ | 1.09±0.056 | 1.09±0.081 | 0.816 |
| λ | 0.50±0.014 | 0.50±0.015 | 0.486 |
| [σ](https://baike.baidu.com/item/σ/7806170) | 0.949±0.055 | 0.946±0.068 | 0.887 |

Data are presented as the mean ± SD

Eglob, global efficiency; Eloc, local efficiency; Cp, clustering coefficient; Lp, shortest path length; γ, normalized clustering coefficient; λ, normalized shortest path length; σ, smallworldness

Table S2 Brain Regions in Anatomical Automatic Labeling atlas (AAL-90)

| **Labels** | **Regions** | **Regions** | **abbr.** | **Anatomical classification** | **x(mm)** | **y(mm)** | **z(mm)** | **voxels within ROIs** |
| --- | --- | --- | --- | --- | --- | --- | --- | --- |
| 1 | Precentral_L | Precental gyrus | PreCG.L | Frontal | -38.65 | -5.68 | 50.94 | 1028 |
| 2 | Precentral_R | Precental gyrus | PreCG.R | Frontal | 41.37 | -8.21 | 52.09 | 1002 |
| 3 | Frontal_Sup_L | Superior frontal gyrus, dorsolateral | SFGdor.L | Prefontal | -18.45 | 34.81 | 42.20 | 1076 |
| 4 | Frontal_Sup_R | Superior frontal gyrus, dorsolateral | SFGdor.R | Prefontal | 21.90 | 31.12 | 43.82 | 1159 |
| 5 | Frontal_Sup_Orb_L | Superior frontal gyrus, orbital part | ORBsup.L | Prefontal | -16.56 | 47.32 | -13.31 | 292 |
| 6 | Frontal_Sup_Orb_R | Superior frontal gyrus, orbital part | ORBsup.R | Prefontal | 18.49 | 48.10 | -14.02 | 311 |
| 7 | Frontal_Mid_L | Middle frontal gyrus | MFG.L | Prefontal | -33.43 | 32.73 | 35.46 | 1448 |
| 8 | Frontal_Mid_R | Middle frontal gyrus | MFG.R | Prefontal | 37.59 | 33.06 | 34.04 | 1510 |
| 9 | Frontal_Mid_Orb_L | Middle frontal gyrus, orbital part | ORBmid.L | Prefontal | -30.65 | 50.43 | -9.62 | 270 |
| 10 | Frontal_Mid_Orb_R | Middle frontal gyrus, orbital part | ORBmid.R | Prefontal | 33.18 | 52.59 | -10.73 | 294 |
| 11 | Frontal_Inf_Oper_L | Inferior frontal gyrus, opercular part | IFGoperc.L | Prefontal | -48.43 | 12.73 | 19.02 | 326 |
| 12 | Frontal_Inf_Oper_R | Inferior frontal gyrus, opercular part | IFGoperc.R | Prefontal | 50.20 | 14.98 | 21.41 | 421 |
| 13 | Frontal_Inf_Tri_L | Inferior frontal gyrus, triangular part | IFGtriang.L | Prefontal | -45.58 | 29.91 | 13.99 | 726 |
| 14 | Frontal_Inf_Tri_R | Inferior frontal gyrus, triangular part | IFGtriang.R | Prefontal | 50.33 | 30.16 | 14.17 | 629 |
| 15 | Frontal_Inf_Orb_L | Inferior frontal gyrus, orbital part | ORBinf.L | Prefontal | -35.98 | 30.71 | -12.11 | 503 |
| 16 | Frontal_Inf_Orb_R | Inferior frontal gyrus, orbital part | ORBinf.R | Prefontal | 41.22 | 32.23 | -11.91 | 505 |
| 17 | Rolandic_Oper_L | Rolandic operculum | ROL.L | Frontal | -47.16 | -8.48 | 13.95 | 302 |
| 18 | Rolandic_Oper_R | Rolandic operculum | ROL.R | Frontal | 52.65 | -6.25 | 14.63 | 399 |
| 19 | Supp_Motor_Area_L | Supplementary motor area | SMA.L | Frontal | -5.32 | 4.85 | 61.38 | 656 |
| 20 | Supp_Motor_Area_R | Supplementary motor area | SMA.R | Frontal | 8.62 | 0.17 | 61.85 | 666 |
| 21 | Olfactory_L | Olfactory cortex | OLF.L | Prefontal | -8.06 | 15.05 | -11.46 | 87 |
| 22 | Olfactory_R | Olfactory cortex | OLF.R | Prefontal | 10.43 | 15.91 | -11.26 | 81 |
| 23 | Frontal_Sup_Medial_L | Superior frontal gyrus, medial | SFGmed.L | Prefontal | -4.80 | 49.17 | 30.89 | 846 |
| 24 | Frontal_Sup_Medial_R | Superior frontal gyrus, medial | SFGmed.R | Prefontal | 9.10 | 50.84 | 30.22 | 641 |
| 25 | Frontal_Mid_Orb_L | Superior frontal gyrus, medial orbital | ORBsupmed.L | Prefontal | -5.17 | 54.06 | -7.40 | 225 |
| 26 | Frontal_Mid_Orb_R | Superior frontal gyrus, medial orbital | ORBsupmed.R | Prefontal | 8.16 | 51.67 | -7.13 | 262 |
| 27 | Rectus_L | Gyrus rectus | REC.L | Prefontal | -5.08 | 37.07 | -18.14 | 261 |
| 28 | Rectus_R | Gyrus rectus | REC.R | Prefontal | 8.35 | 35.64 | -18.04 | 218 |
| 29 | Insula_L | Insula | INS.L | Subcortical | -35.13 | 6.65 | 3.44 | 566 |
| 30 | Insula_R | Insula | INS.R | Subcortical | 39.02 | 6.25 | 2.08 | 539 |
| 31 | Cingulum_Ant_L | Anterior cingulate and paracingulate gyri | ACG.L | Prefontal | -4.04 | 35.40 | 13.95 | 426 |
| 32 | Cingulum_Ant_R | Anterior cingulate and paracingulate gyri | ACG.R | Prefontal | 8.46 | 37.01 | 15.84 | 397 |
| 33 | Cingulum_Mid_L | Median cingulate and paracingulate gyri | DCG.L | Frontal | -5.48 | -14.92 | 41.57 | 619 |
| 34 | Cingulum_Mid_R | Median cingulate and paracingulate gyri | DCG.R | Frontal | 8.02 | -8.83 | 39.79 | 605 |
| 35 | Cingulum_Post_L | Posterior cingulate gyrus | PCG.L | Parietal | -4.85 | -42.92 | 24.67 | 137 |
| 36 | Cingulum_Post_R | Posterior cingulate gyrus | PCG.R | Parietal | 7.44 | -41.81 | 21.87 | 87 |
| 37 | Hippocampus_L | Hippocampus | HIP.L | Temporal | -25.03 | -20.74 | -10.13 | 273 |
| 38 | Hippocampus_R | Hippocampus | HIP.R | Temporal | 29.23 | -19.78 | -10.33 | 288 |
| 39 | ParaHippocampal_L | Parahippocampal gyrus | PHG.L | Temporal | -21.17 | -15.95 | -20.70 | 286 |
| 40 | ParaHippocampal_R | Parahippocampal gyrus | PHG.R | Temporal | 25.38 | -15.15 | -20.47 | 316 |
| 41 | Amygdala_L | Amygdala | AMYG.L | Temporal | -23.27 | -0.67 | -17.14 | 62 |
| 42 | Amygdala_R | Amygdala | AMYG.R | Temporal | 27.32 | 0.64 | -17.50 | 70 |
| 43 | Calcarine_L | Calcarine fissure and surrounding cortex | CAL.L | Occipital | -7.14 | -78.67 | 6.44 | 648 |
| 44 | Calcarine_R | Calcarine fissure and surrounding cortex | CAL.R | Occipital | 15.99 | -73.15 | 9.40 | 542 |
| 45 | Cuneus_L | Cuneus | CUN.L | Occipital | -5.93 | -80.13 | 27.22 | 449 |
| 46 | Cuneus_R | Cuneus | CUN.R | Occipital | 13.51 | -79.36 | 28.23 | 434 |
| 47 | Lingual_L | Lingual gyrus | LING.L | Occipital | -14.62 | -67.56 | -4.63 | 660 |
| 48 | Lingual_R | Lingual gyrus | LING.R | Occipital | 16.29 | -66.93 | -3.87 | 678 |
| 49 | Occipital_Sup_L | Superior occipital gyrus | SOG.L | Occipital | -16.54 | -84.26 | 28.17 | 396 |
| 50 | Occipital_Sup_R | Superior occipital gyrus | SOG.R | Occipital | 24.29 | -80.85 | 30.59 | 428 |
| 51 | Occipital_Mid_L | Middle occipital gyrus | MOG.L | Occipital | -32.39 | -80.73 | 16.11 | 959 |
| 52 | Occipital_Mid_R | Middle occipital gyrus | MOG.R | Occipital | 37.39 | -79.70 | 19.42 | 595 |
| 53 | Occipital_Inf_L | Inferior occipital gyrus | IOG.L | Occipital | -36.36 | -78.29 | -7.84 | 268 |
| 54 | Occipital_Inf_R | Inferior occipital gyrus | IOG.R | Occipital | 38.16 | -81.99 | -7.61 | 314 |
| 55 | Fusiform_L | Fusiform gyrus | FFG.L | Temporal | -31.16 | -40.30 | -20.23 | 687 |
| 56 | Fusiform_R | Fusiform gyrus | FFG.R | Temporal | 33.97 | -39.10 | -20.18 | 760 |
| 57 | Postcentral_L | Postcentral gyrus | PoCG.L | Parietal | -42.46 | -22.63 | 48.92 | 1159 |
| 58 | Postcentral_R | Postcentral gyrus | PoCG.R | Parietal | 41.43 | -25.49 | 52.55 | 1138 |
| 59 | Parietal_Sup_L | Superior parietal gyrus | SPG.L | Parietal | -23.45 | -59.56 | 58.96 | 631 |
| 60 | Parietal_Sup_R | Superior parietal gyrus | SPG.R | Parietal | 26.11 | -59.18 | 62.06 | 647 |
| 61 | Parietal_Inf_L | Inferior parietal, but supramarginal and angular gyri | IPL.L | Parietal | -42.80 | -45.82 | 46.74 | 696 |
| 62 | Parietal_Inf_R | Inferior parietal, but supramarginal and angular gyri | IPL.R | Parietal | 46.46 | -46.29 | 49.54 | 419 |
| 63 | SupraMarginal_L | Supramarginal gyrus | SMG.L | Parietal | -55.79 | -33.64 | 30.45 | 354 |
| 64 | SupraMarginal_R | Supramarginal gyrus | SMG.R | Parietal | 57.61 | -31.50 | 34.48 | 550 |
| 65 | Angular_L | Angular gyrus | ANG.L | Parietal | -44.14 | -60.82 | 35.59 | 342 |
| 66 | Angular_R | Angular gyrus | ANG.R | Parietal | 45.51 | -59.98 | 38.63 | 496 |
| 67 | Precuneus_L | Precuneus | PCUN.L | Parietal | -7.24 | -56.07 | 48.01 | 1079 |
| 68 | Precuneus_R | Precuneus | PCUN.R | Parietal | 9.98 | -56.05 | 43.77 | 935 |
| 69 | Paracentral_Lobule_L | Paracentral lobule | PCL.L | Parietal | -7.63 | -25.36 | 70.07 | 422 |
| 70 | Paracentral_Lobule_R | Paracentral lobule | PCL.R | Parietal | 7.48 | -31.59 | 68.09 | 227 |
| 71 | Caudate_L | Caudate nucleus | CAU.L | Subcortical | -11.46 | 11.00 | 9.24 | 278 |
| 72 | Caudate_R | Caudate nucleus | CAU.R | Subcortical | 14.84 | 12.07 | 9.42 | 284 |
| 73 | Putamen_L | Lenticular nucleus, putamen | PUT.L | Subcortical | -23.91 | 3.86 | 2.40 | 306 |
| 74 | Putamen_R | Lenticular nucleus, putamen | PUT.R | Subcortical | 27.78 | 4.91 | 2.46 | 322 |
| 75 | Pallidum_L | Lenticular nucleus, pallidum | PAL.L | Subcortical | -17.75 | -0.03 | 0.21 | 81 |
| 76 | Pallidum_R | Lenticular nucleus, pallidum | PAL.R | Subcortical | 21.20 | 0.18 | 0.23 | 76 |
| 77 | Thalamus_L | Thalamus | THA.L | Subcortical | -10.85 | -17.56 | 7.98 | 313 |
| 78 | Thalamus_R | Thalamus | THA.R | Subcortical | 13.00 | -17.55 | 8.09 | 307 |
| 79 | Heschl_L | Heschl gyrus | HES.L | Temporal | -41.99 | -18.88 | 9.98 | 72 |
| 80 | Heschl_R | Heschl gyrus | HES.R | Temporal | 45.86 | -17.15 | 10.41 | 73 |
| 81 | Temporal_Sup_L | Superior temporal gyrus | STG.L | Temporal | -53.16 | -20.68 | 7.13 | 672 |
| 82 | Temporal_Sup_R | Superior temporal gyrus | STG.R | Temporal | 58.15 | -21.78 | 6.80 | 963 |
| 83 | Temporal_Pole_Sup_L | Temporal pole: superior temporal gyrus | TPOsup.L | Temporal | -39.88 | 15.14 | -20.18 | 382 |
| 84 | Temporal_Pole_Sup_R | Temporal pole: superior temporal gyrus | TPOsup.R | Temporal | 48.25 | 14.75 | -16.86 | 400 |
| 85 | Temporal_Mid_L | Middle temporal gyrus | MTG.L | Temporal | -55.52 | -33.80 | -2.20 | 1439 |
| 86 | Temporal_Mid_R | Middle temporal gyrus | MTG.R | Temporal | 57.47 | -37.23 | -1.47 | 1356 |
| 87 | Temporal_Pole_Mid_L | Temporal pole: middle temporal gyrus | TPOmid.L | Temporal | -36.32 | 14.59 | -34.08 | 222 |
| 88 | Temporal_Pole_Mid_R | Temporal pole: middle temporal gyrus | TPOmid.R | Temporal | 44.22 | 14.55 | -32.23 | 349 |
| 89 | Temporal_Inf_L | Inferior temporal gyrus | ITG.L | Temporal | -49.77 | -28.05 | -23.17 | 941 |
| 90 | Temporal_Inf_R | Inferior temporal gyrus | ITG.R | Temporal | 53.69 | -31.07 | -22.32 | 1072 |

**References**

Achard, S., and Bullmore, E. (2007). Efficiency and cost of economical brain functional networks. *PLoS Comput Biol* 3(2)**,** e17. doi: 10.1371/journal.pcbi.0030017.

Buckner, R.L., Sepulcre, J., Talukdar, T., Krienen, F.M., Liu, H., Hedden, T., et al. (2009). Cortical hubs revealed by intrinsic functional connectivity: mapping, assessment of stability, and relation to Alzheimer's disease. *J Neurosci* 29(6)**,** 1860-1873. doi: 10.1523/JNEUROSCI.5062-08.2009.

Latora, V., and Marchiori, M. (2001). Efficient behavior of small-world networks. *Phys Rev Lett* 87(19)**,** 198701.

Liao, X.H., Xia, M.R., Xu, T., Dai, Z.J., Cao, X.Y., Niu, H.J., et al. (2013). Functional brain hubs and their test-retest reliability: A multiband resting-state functional MRI study. *Neuroimage* 83**,** 969-982. doi: 10.1016/j.neuroimage.2013.07.058.

Maslov, S., and Sneppen, K. (2002). Specificity and stability in topology of protein networks. *Science* 296(5569)**,** 910-913. doi: 10.1126/science.1065103.

Onnela, J.P., Saramaki, J., Kertesz, J., and Kaski, K. (2005). Intensity and coherence of motifs in weighted complex networks. *Phys Rev E Stat Nonlin Soft Matter Phys* 71(6 Pt 2)**,** 065103.

Sporns, O., and Zwi, J.D. (2004). The small world of the cerebral cortex. *Neuroinformatics* 2(2)**,** 145-162. doi: 10.1385/NI:2:2:145.

Tzourio-Mazoyer, N., Landeau, B., Papathanassiou, D., Crivello, F., Etard, O., Delcroix, N., et al. (2002). Automated anatomical labeling of activations in SPM using a macroscopic anatomical parcellation of the MNI MRI single-subject brain. *Neuroimage* 15(1)**,** 273-289. doi: 10.1006/nimg.2001.0978.

Watts, D.J., and Strogatz, S.H. (1998). Collective dynamics of 'small-world' networks. *Nature* 393(6684)**,** 440-442. doi: Doi 10.1038/30918.

Zuo, X.N., Ehmke, R., Mennes, M., Imperati, D., Castellanos, F.X., Sporns, O., et al. (2012). Network centrality in the human functional connectome. *Cereb Cortex* 22(8)**,** 1862-1875. doi: 10.1093/cercor/bhr269.
